# Therapeutic effects and potential mechanisms of astragaloside IV on pulmonary fibrosis: a systematic review and meta-analysis of preclinical studies

**DOI:** 10.3389/fphar.2025.1564290

**Published:** 2025-07-31

**Authors:** Shujuan Zhang, Yan Xue, Xing Zhang, Feng Chen, Yalan Li, Wei Zhang

**Affiliations:** ^1^Department of Pulmonary Diseases, Shuguang Hospital, Shanghai University of Traditional Chinese Medicine, Shanghai, China; ^2^ Shanghai University of Traditional Chinese Medicine, Shanghai, China; ^3^Zhang Wei Baoshan Famous Traditional Chinese Medicine Inheritance Studio, Shanghai Baoshan Hospital of Integrated Chinese and Western Medicine, Shanghai, China; ^4^Institute of Infectious Diseases, Shanghai Institute of Traditional Chinese Medicine, Shanghai, China

**Keywords:** Astragalus mongholicus, astragaloside IV, animal model, pulmonary fibrosis, meta-analysis, systematic review

## Abstract

**Background:**

Pulmonary fibrosis (PF) remains a devastating disease with limited therapeutic options. Astragaloside IV (AS-IV), a natural compound from Astragalus mongholicus (AM), has shown promise as a possible treatment for fibrosis. However, a systematic evaluation of its therapeutic efficacy and underlying mechanisms is lacking. This meta-analysis synthesizes preclinical evidence to assess the therapeutic potential of AS-IV in PF.

**Methods:**

Preclinical literature published before 16 August 2024, was systematically retrieved and screened across eight major databases, including PubMed, Embase, Web of Science, Cochrane Library, China National Knowledge Infrastructure (CNKI), Wan Fang Data Knowledge Service Platforms (Wanfang), China Science and Technology Journal Database (CQVIP), and China Biological Medicine Database (CBM). The risk of bias was assessed using the Systematic Review Centre for Laboratory Animal Experimentation (SYRCLE) tool, and meta-analysis was conducted using STATA 18.0. The underlying mechanisms were also summarized.

**Results:**

This systematic review and meta-analysis encompassed 23 *in vivo* animal studies comprising a total of 518 animals. The methodological quality scores of the included studies ranged from 3 to 6 points. The overall analysis demonstrated that AS-IV significantly reduced key indicators of PF in animal models, including PF score [SMD = −2.56, 95% CI (−3.47, −1.65), *P <* 0.01, *I* ^*2*^ *=* 72.6%]; pulmonary inflammation scores [SMD = −2.18, 95% CI (−3.09, −1.27), *P <* 0.01, *I* ^*2*^ *=* 70.2%]; hydroxyproline (HYP) content [SMD = −4.31, 95% CI (−5.67, −2.95), *P <* 0.01, *I* ^*2*^ *=* 83.1%]; lung index [SMD = −3.43, 95% CI (−4.75, −2.10), *P <* 0.01, *I* ^*2*^ *=* 79.5%]; and α-smooth muscle actin (α-SMA) levels [SMD = −4.79, 95% CI (−6.01, −3.56), *P <* 0.01, *I* ^*2*^ *=* 55.3%]. Sensitivity analyses confirmed the robustness of these results. However, the asymmetry observed in the funnel plot suggests potential publication bias. Further analysis revealed that AS-IV modulates key biomarkers involved in the epithelial-mesenchymal transition (EMT) process and mitigates extracellular matrix (ECM) remodeling. Additionally, AS-IV reduces the levels of inflammatory markers and oxidative stress indicators, thereby exerting a significant intervention in PF.

**Conclusion:**

This meta-analysis demonstrates that AS-IV consistently ameliorates BLM-induced PF through multiple mechanisms, including inhibition of EMT, ECM remodeling, inflammation, and oxidative stress. These findings support further investigation of AS-IV as a multi-target therapeutic agent for PF.

**Systematic Review Registration:**

identifier CRD42024604432.

## 1 Introduction

Pulmonary fibrosis (PF) is a chronic, progressive, and irreversible lung disease characterized by pathological extracellular matrix (ECM) deposition, which leads to the deterioration of respiratory function and markedly reduced patient survival (Martinez et al., 2017; Spagnolo et al., 2021). The pathogenesis of PF is complex and multifactorial, involving aging, genetic susceptibility, and environmental exposures. These factors converge to cause repeated injury to the alveolar epithelium, disrupt cellular metabolism, and impair tissue repair, ultimately leading to progressive fibrosis (Heukels et al., 2019; Puglisi et al., 2016). This initial epithelial damage triggers a cascade of pathological events, including epigenetic modifications and epithelial-mesenchymal transition (EMT), which promote the activation of fibroblasts and the production of uncontrolled ECM (Duong and Hagood, 2018). These interconnected processes culminate in the irreversible architectural destruction of the lung tissue, which defines PF (Moss et al., 2022).

The clinical and social effects of PF are significant. Epidemiological studies have demonstrated a striking age-related increase in the disease’s incidence (Nalysnyk et al., 2012). As the global population continues to age, the socioeconomic burden of PF is projected to escalate dramatically in the coming decades (Raghu et al., 2014). Despite this growing challenge, current therapeutic options remain disappointingly inadequate and are hampered by their modest efficacy and significant adverse effects (Dempsey et al., 2019; Noble et al., 2016). This therapeutic gap highlights the urgent need for innovative treatment approaches targeting the fundamental molecular mechanisms underlying fibrotic progression.

Traditional Chinese Medicine (TCM) has demonstrated distinct therapeutic advantages in the prevention and treatment of PF (Geng et al., 2024). The TCM therapeutic philosophy of “holistic regulation and multi-target intervention” aligns remarkably well with the multifactorial pathogenesis and multi-stage pathological progression characteristic of PF. According to the classical TCM theory of “qi governing defense,” the pathological essence of PF can be attributed to impaired lung defense function, leading to “accumulation of turbid toxins,” with treatment following the fundamental principle of “tonifying the qi to consolidate the foundation” (Li J. et al., 2022; Societies, 2025). Astragalus mongholicus (AM), a quintessential qi-tonifying herb in TCM, serves as the principal therapeutic component in classical antifibrotic formulations, including Huangqi Guizhi Wuwu Decoction, Danggui Buxue Decoction, and Buyang Huanwu Decoction. Contemporary data mining analyses of clinical prescriptions have demonstrated that AM exhibits the highest utilization frequency among all herbs prescribed for PF treatment, substantiating its pivotal role in the TCM therapeutic framework for this disease (Gao et al., 2011; Zhang et al., 2018).

Astragaloside IV (AS-IV), the predominant bioactive saponin isolated from AM, is a cycloastragenol-type tetracyclic triterpenoid (Sun et al., 2016). This compound has been established as a critical quality marker for Astragalus preparations in the Chinese Pharmacopoeia (Li et al., 2023). AS-IV exhibits pleiotropic pharmacological activities, including anti-inflammatory, immunomodulatory, antioxidant, and antifibrotic properties as well as cardioprotective, antimicrobial, and antiviral effects (Zhang et al., 2020). This multifaceted pharmacological profile establishes AS-IV as a compelling therapeutic candidate with broad clinical applications across diverse organ systems. Accumulating evidence indicates that AS-IV represents the primary pharmacological basis underlying the antifibrotic effects of AM via multiple molecular mechanisms (Liang et al., 2023). Despite the exponential growth in preclinical studies investigating AS-IV (Zhu et al., 2024), the existing research remains fragmented and consists mainly of isolated experimental investigations without a systematic, evidence-based evaluation. This methodological limitation substantially impedes the scientific assessment of AS-IV’s true translational potential. Systematic reviews and meta-analyses of animal models are indispensable tools for objectively evaluating therapeutic efficacy and guiding the clinical translation of candidate compounds (Ineichen et al., 2024; van Luijk et al., 2013).

This study aimed to evaluate the therapeutic efficacy and mechanistic pathways of AS-IV in bleomycin-induced PF animal models through a comprehensive systematic review and meta-analysis. By quantitatively analyzing AS-IV’s modulatory effects on key fibrotic biomarkers, we aimed to establish evidence-based support for the clinical translation of AS-IV in PF therapy, accelerate the progression from preclinical studies to human clinical trials, and provide mechanistic insights for the development of novel antifibrotic therapeutics.

## 2 Materials and methods

This systematic review was conducted following the Preferred Reporting Items for Systematic Reviews and Meta-Analyses (PRISMA) guidelines (Page et al., 2021). The risk of bias was assessed using the SYstematic Review Centre for Laboratory animal Experimentation (SYRCLE) tool for animal intervention studies (Hooijmans et al., 2014). The protocol was registered in the PROSPERO database (registration number: CRD42024604432) before conducting the review.

### 2.1 Search strategies

Two investigators independently conducted comprehensive searches across eight electronic databases: PubMed, Embase, Web of Science, Cochrane Library, China National Knowledge Infrastructure (CNKI), Wan Fang Data Knowledge Service Platforms (Wanfang), China Science and Technology Journal Database (CQVIP), and China Biological Medicine Database (CBM) to identify relevant *in vivo* animal studies published from the establishment of the databases up to 16 August 2024. The search strategy combined Medical Subject Headings (MeSH) terms with free-text keywords, adapted for each database’s specific indexing system and search capabilities, without language or publication date restrictions. The PubMed database was searched using the following terms: “Pulmonary Fibrosis” OR “Fibroses, Pulmonary” OR “Fibrosis, Pulmonary” OR “Pulmonary Fibroses” OR “Alveolitis, Fibrosing” OR “Alveolitides, Fibrosing” OR “Fibrosing Alveolitides” OR “Fibrosing Alveolitis” OR “Idiopathic Diffuse Interstitial Pulmonary Fibrosis” AND “astragaloside A″ OR “astragaloside-A″ OR “astragaloside IV” OR “astramembrannin I″ OR “cyclosiversioside F″ OR ″3beta,6alpha,16beta,20R,24S: astragaloside IV of astragaloside A”. Supplementary Table S1 provides details of the search strategy for PubMed.

### 2.2 Inclusion and exclusion criteria

Inclusion criteria were established according to the PICO framework: (1) Participants: *in vivo* animal models of PF induced by bleomycin (BLM), a model demonstrating robust reproducibility that accurately recapitulates key pathophysiological features of human PF (Jenkins et al., 2017); (2) Intervention: AS-IV administered as a single compound with clearly defined dosage, route of administration, and treatment duration; (3) Comparison: control groups comprising BLM-induced PF models receiving either vehicle treatment or no treatment; (4) Outcomes: Primary outcome measures encompassed PF score, pulmonary inflammation score, lung index, hydroxyproline (HYP) content, and α-smooth muscle actin (α-SMA) expression. Secondary outcomes included EMT biomarkers—specifically transforming growth factor-β1 (TGF-β1) mRNA and protein expression and E-cadherin levels. ECM remodeling was assessed through the expression of collagen types I and III at both mRNA and protein levels. Additional parameters comprised lung wet-to-dry weight ratio for edema assessment, oxidative stress markers (superoxide dismutase [SOD] activity, malondialdehyde [MDA] and reactive oxygen species [ROS] levels), and inflammatory cytokines (tumor necrosis factor-α [TNF-α], interleukin-6 [IL-6], interleukin-18 [IL-18], and interleukin-1β [IL-1β]). These comprehensive biomarkers facilitate a multidimensional evaluation of PF pathogenesis and therapeutic response.

Exclusion criteria included: (1) *in vitro* studies, clinical trials, systematic reviews, meta-analyses, or case reports; (2) studies using AS-IV in combination with other active compounds; (3) inappropriate or incompletely described control groups; (4) non-PF models or PF models not induced by BLM; (5) absence of extractable data for predefined outcomes.

### 2.3 Data extraction

The retrieved literature was managed using EndNote X9. Following deduplication, two researchers independently screened studies according to predefined inclusion and exclusion criteria, with subsequent cross-validation. Discrepancies were resolved through consultation with a third researcher until consensus was achieved. Data extraction was performed using a standardized Excel 2019 template, capturing (1) publication details (authors, year); (2) intervention characteristics (administration route, treatment duration); (3) BLM induction protocols; (4) animal characteristics (species, strain, sex, age, weight); and (5) outcome measures (e.g., PF and pulmonary inflammation scores, biomarker expression including HYP and α-SMA).

Given the substantial heterogeneity in dosing regimens across studies, we adopted a systematic approach to address this methodological challenge. For studies evaluating multiple AS-IV doses, we extracted data from the group receiving the highest therapeutically effective dose. This approach was implemented to assess the maximum therapeutic potential of AS-IV against PF, providing insights into optimal dosing strategies for potential clinical translation. By analyzing peak therapeutic responses, our meta-analysis offers evidence for the upper bounds of AS-IV efficacy. All outcomes were continuous variables. Means, standard deviations (SD), and sample sizes (n) were extracted directly when available. For data presented exclusively in graphical format, we first contacted the corresponding authors to request raw data. When unavailable, WebPlotDigitizer (version 4.5) was employed for data extraction following validated protocols.

### 2.4 Bias assessment

Methodological quality was independently evaluated by two researchers using the SYRCLE risk of bias tool, which assesses the following domains: (1) Was the allocation sequence adequately generated and applied? (2) Were the groups similar at baseline, or were they adjusted for confounders in the analysis? (3) Was the allocation adequately concealed? (4) Were the animals randomly housed during the experiment? (5) Were the caregivers and/or investigators blinded to which intervention each animal received during the experiment? (6) Were animals selected at random for outcome assessment? (7) Was the outcome assessor blinded? (8) Were incomplete outcome data adequately addressed? (9) Are the reports of the study free from selective outcome reporting? (10) Was the study free from other issues that could result in a high risk of bias? The evaluation criteria were as follows: a response of “yes” indicated low risk, “no” indicated high risk, and “unclear” indicated insufficient information to assess the risk of bias.

### 2.5 Statistical analysis

Data analysis and visualization were performed using Stata 18.0. All outcome measures were continuous variables, with effect sizes calculated as standardized mean differences (SMD) and 95% confidence intervals (CIs). SMD was selected to accommodate the varying measurement scales across studies. A *p-value* of <0.05 was considered statistically significant. Heterogeneity was evaluated using the *I* ^2^ statistic and Cochran’s *Q* test. An *I* ^2^ > 50% or *P <* 0.1 indicated substantial heterogeneity, warranting the application of a random-effects model; otherwise, a fixed-effects model was employed. For outcomes demonstrating substantial heterogeneity, sensitivity analyses were performed by sequentially excluding individual studies to assess the robustness and stability of the pooled results. If a study significantly influenced heterogeneity, the effect size was recalculated after its exclusion. When substantial heterogeneity in primary outcomes could not be explained through sensitivity analyses, subgroup analyses were performed to identify potential sources of heterogeneity. In this study, subgroup analyses were conducted based on rodent species (rats vs mice) and treatment duration, as these factors were considered potential contributors to heterogeneity. Furthermore, for analyses involving ten or more included studies, funnel plots and Egger’s test were conducted using Stata 18.0 to assess the presence of publication bias. Statistical significance was set at *P <* 0.05.

## 3 Results

### 3.1 Characteristics of included studies

**FIGURE 1:**
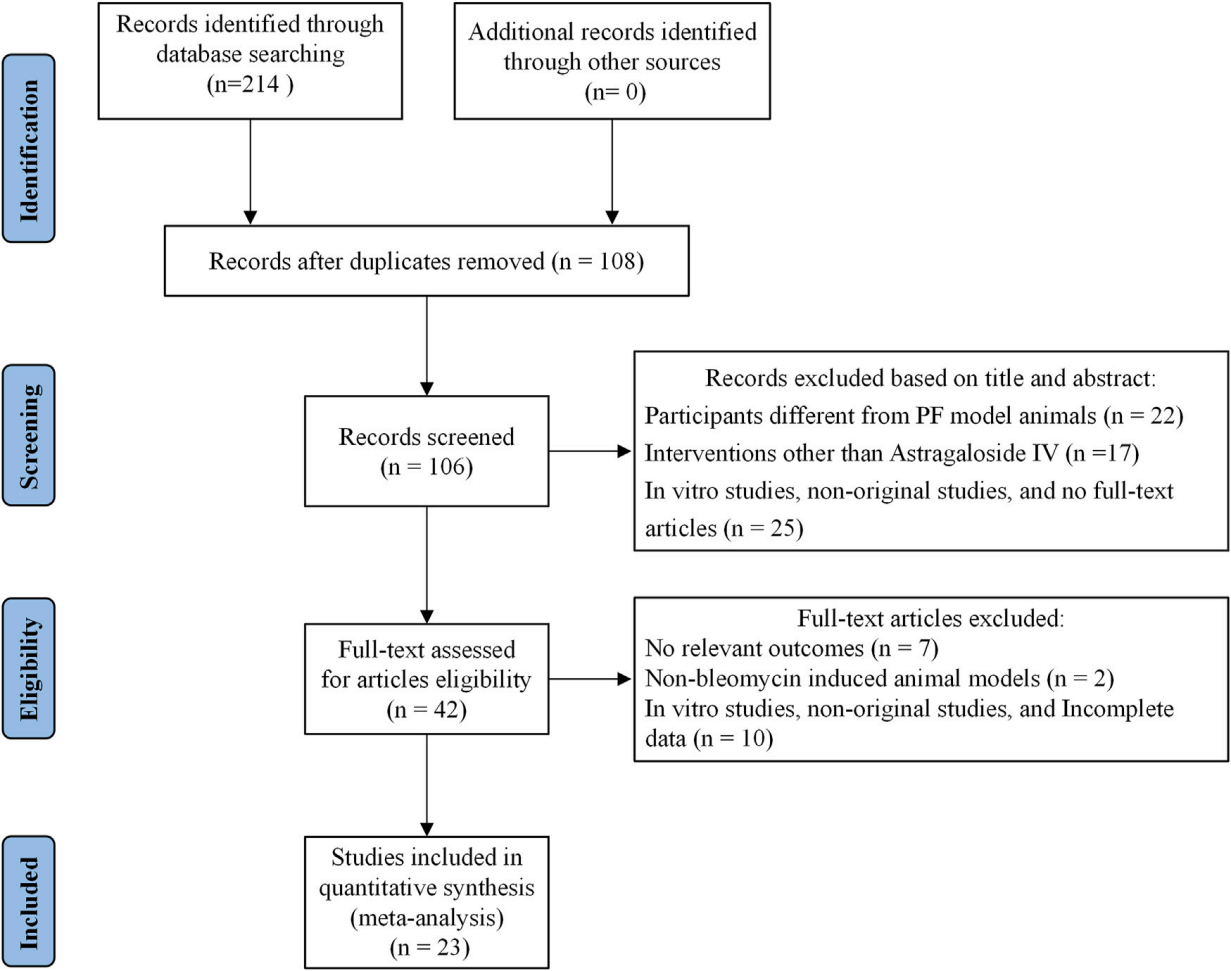
The flowchart of the studies identification and inclusion process.

This systematic review and meta-analysis synthesized data from 23 *in vivo* studies comprising 518 animals with BLM-induced PF, equally distributed between the AS-IV treatment group (n = 259) and the control group (n = 259). The detailed literature screening process is shown in Figure 1. The included studies encompassed two rodent species: mice (12 studies, including C57BL/6J, Kunming, and C57BL substrains) and rats (11 studies, predominantly Sprague-Dawley and Wistar strains). This taxonomic diversity enhances the generalizability of findings across different genetic backgrounds.

The included studies exhibited considerable heterogeneity in AS-IV administration protocols, characteristic of exploratory preclinical investigations. Dosing regimens ranged from 1 to 200 mg/kg/day, delivered via multiple routes, including oral gavage (predominant), intratracheal instillation, and nebulized inhalation. This diversity in administration routes reflects attempts to optimize bioavailability and therapeutic efficacy for potential clinical translation. Treatment durations varied from 14 to 28 days, consistent with established timeframes for evaluating antifibrotic efficacy in rodent models of PF.

While methodological heterogeneity increases analytical complexity, it paradoxically establishes a multidimensional evidence map that offers actionable insights for optimizing therapeutic protocols in the translation from bench to bedside. To accommodate this variability and enhance the robustness of our findings, we implemented random-effects meta-analytical models. Additionally, we performed stratified subgroup analyses by species (mice vs rats) and treatment duration (28 days vs <28 days) to investigate potential moderators of treatment effect and assess the consistency of AS-IV’s antifibrotic efficacy across different experimental conditions.

Primary outcomes included histopathological assessments (PF score: 10 studies; pulmonary inflammation score: nine studies), biochemical markers (HYP content: 14 studies), physiological parameters (lung index: eight studies), and myofibroblast activation markers (α-SMA expression: 11 studies). Secondary outcomes were categorized according to key pathophysiological processes. EMT markers included TGF-β1 mRNA (n = 4), TGF-β1 protein (n = 9), and E-cadherin expression (n = 5). ECM remodeling was assessed through collagen type I mRNA (n = 3), collagen type I protein (n = 4), and collagen type III protein (n = 3). Oxidative stress parameters included SOD activity (n = 5), MDA levels (n = 6), and ROS (n = 4). Inflammatory mediators included TNF-α (n = 6), IL-6 (n = 6), IL-18 (n = 2), and IL-1β (n = 3). Additionally, pulmonary edema was evaluated using lung wet-to-dry weight ratios (n = 3).

Although several secondary outcomes were derived from a limited number of studies, their inclusion was deemed essential for elucidating the pleiotropic mechanisms underlying AS-IV’s antifibrotic activity. We acknowledge that analyses based on fewer studies may have reduced statistical power; however, this comprehensive approach provides valuable insights into the multifaceted pharmacological actions of AS-IV in PF. Detailed study characteristics and outcome measurements are summarized in Table 1.

**TABLE 1:** Basic characteristics of the included studies.

Study	Species (strain; sex; age; weight)	Sample (T/M)	Modeling method	Anesthesia method	Intervention	Outcomes	Mechanisms
Xu et al. (2017)	Mouse (C57BL/6; Male; 6 weeks; 18–20 g)	15/15	Intratracheal injection BLM 10 mg/kg	Intraperitoneal injection, 4% chloral hydrate 10 mL/kg	Oral gavage 100 mg/kg, daily; 28 days	①⑥⑩⑬⑭⑰	1. Suppression of inflammation 2. Inhibiting activation of fibrosis-associated fibroblasts 3. Enhancing autophagy
Wang et al. (2018)	Mouse (C57BL/6; Male and Female; 6 weeks; 18–20 g)	20/20	Intratracheal instillation, BLM 10 mg/kg	Intraperitoneal injection, 4% chloral hydrate 10 mL/kg	Oral gavage 50 mg/kg, daily; 14 days	⑦⑧	1. Enhancement of autophagy activity 2. Reduction of pyroptosis-related protein expression 3. Suppression of inflammation
Han et al. (2019)	Mouse (Kunming; Male; 6 weeks; 18 ± 2 g)	15/15	Intratracheal injection BLM 10 mg/kg	Intraperitoneal injection, 4% chloral hydrate 10 mg/kg	Oral gavage 40 mg/kg, daily; 28 days	①⑤⑥⑩⑬⑭	Inhibition of EMT.
Xie (2016)	Rat (Wistar; Male and Female; NM; 200 ± 20 g)	7/7	Intratracheal injection BLM 5 mg/kg	Intraperitoneal injection,10% chloral hydrate 10 mL/kg	Oral gavage 200 mg/kg daily; 28 days	⑩⑬	Inhibition of pathological angiogenesis
Xiao (2009)	Rat (Sprague Dawley; Male and Female; 3–4 months; 180–230 g)	6/6	Intratracheal injection BLM 5 mg/kg	Intraperitoneal injection, 2% pentobarbital sodium, 45 mg/kg	Oral gavage 1 mg/kg, daily; 28 days	⑥⑬⑭	Inhibition of excessive expression of migration inhibitory factor
Wang et al. (2012)	Rat (Sprague Dawley; Male; NM; 250 ± 30 g)	6/6	Intratracheal instillation, BLM 5 mg/kg	Intraperitoneal injection 10% chloral hydrate 3 mL/kg	Oral gavage 36 mg/kg, daily; 28 days	⑥⑬⑰⑱	1. Inhibition of fibroblast activation 2. Attenuation of collagen deposition 3. Suppression of inflammation
Liu et al. (2015)	Mouse (C57BL/6J; Male; 8 weeks; 20–22 g)	5/5	Intratracheal injection BLM 3 mg/kg	NM	Oral gavage 100 mg/kg, daily; 21 daysay	⑥⑫⑬⑭⑰	Regulation of oxidative stress
Lin (2022)	Rat (Sprague Dawley; Male; NM; 200 ± 20 g)	8/8	Intratracheal instillation, BLM 5 mg/kg	Intraperitoneal injection 10% chloral hydrate 3.0 mL/kg	Oral gavage 200 mg/kg, daily; 28 days	①⑤⑥⑨⑩⑰⑱⑲	1. Inhibition of fibroblast activation 2. Suppression of inflammation
Huang (2019)	Mouse (C57BL/6; Male and Female; 6 weeks; 18–20 g)	5/5	Intratracheal injection BLM 10 mL/kg	Intraperitoneal injection, 4% chloral hydrate 10 mL/kg	Oral gavage 60 mg/kg daily; 28 days	①②④⑥	1. Inhibition of fibroblast proliferation and activation 2. Suppression of collagen deposition
Zhang et al. (2007)	Rat (Sprague Dawley; NM; NM; 180–230 g)	6/6	Intratracheal injection BLM 5 mg/kg	Intraperitoneal injection, 2% pentobarbital sodium, 45 mg/kg	Oral gavage 1 mg/kg, daily; 28 days	⑥	Inhibition of excessive Cathepsin B expression
Li (2007)	Rat (Sprague Dawley; Male and Female; 3–4 months; 180–230 g)	6/6	Intratracheal injection BLM 5 mg/kg	Intraperitoneal injection, 2% pentobarbital sodium, 45 mg/kg	Oral gavage 1 mg/kg, daily; 28 days	⑥⑬⑭	Inhibition of excessive Cathepsin B expression
Tong et al. (2021a)	Mouse (C57BL; Male; NM; 22 ± 2 g)	8/8	Intratracheal instillation, BLM 2 U/kg	Isoflurane	Oral gavage 40.8 mg/kg, daily; 28 days	⑥⑫⑮⑯⑰⑲	1. Inhibition of fibroblast proliferation and collagen deposition 2. Regulation of oxidative stress
Miao (2015)	Rat (Wistar; Male and Female; NM; 200 ± 20 g)	21/21	Intratracheal injection BLM 5 mg/kg	Intraperitoneal injection 10% chloral hydrate 3 mL/kg	Oral gavage 200 mg/kg, daily; 28 days	⑬⑭	1. Inhibition of angiogenesis 2. Suppression of inflammation
Li et al. (2024)	Mouse (C57BL/6; NM; NM; NM)	10/10	Intratracheal injection BLM 10 mg/kg	NM	Oral gavage 40 mg/kg daily 28 d	①②③⑨⑪⑱	Autophagy activation
Yu et al. (2016)	Rat (Sprague Dawley; Male; 8 weeks; 250 g)	10/10	Intratracheal instillation, BLM 5 mg/kg	10% chloral hydrate 3.5 mL/kg	Intraperitoneal injection 50 mg/kg, daily; 28 days	⑧⑨⑪⑫⑮⑯⑱	Inhibiting oxidative stress and inflammatory response
Yu et al. (2022)	Mouse (Kunming; Male; 4 weeks; 18–22 g)	6/6	Intratracheal injection BLM 5 mg/kg	NM	Oral gavage 200 mg/kg, daily; 28 days	①②③④⑥⑩⑭	Modulating autophagy
Qian et al. (2018)	Rat (NM; NM; NM; NM)	5/5	Intratracheal instillation BLM 5 mg/kg	Intraperitoneal injection 10% chloral hydrate 5 mL/kg	Oral gavage 20 mg/kg, daily; 14 days	①②③⑤⑨⑪⑫⑯⑰⑱⑲	Inhibition of EMT.
Li et al. (2017)	Rat (Sprague Dawley; Female; Adult; NM)	10/10	Intratracheal instillation, BLM 5 mg/kg	Intraperitoneal injection 10% chloral hydrate 2.5 mL/kg	Oral gavage 10 mg/kg, daily; 28 d	①④⑥⑩⑬⑭	Inhibition of EMT.
Guan et al. (2024)	Rat (Sprague Dawley; Male; NM; 160–180 g)	7/7	Intratracheal instillation, BLM 5 mg/kg	NM	Oral gavage 20 mg/kg, daily; 14 days	①⑤	Inhibition of EMT.
Huang et al. (2019)	Mouse (C57BL/6; Male; 6 weeks; 18–20 g)	10/10	Intratracheal instillation, BLM 10 mg/kg	Intraperitoneal injection, 4% chloral hydrate 10 mL/kg	Oral gavage 50 mg/kg, daily; 14 days	⑩⑫⑬⑭⑮⑯	Modulating autophagy and oxidative stress
Tong et al. (2021b)	Mouse (C57BL; Male; NM; 22–24 g)	10/10	Intratracheal instillation, BLM 2 U/kg	Isoflurane solution NM	Intratracheal administration 40.8 mg/kg, every 3 days; 28 days	⑥⑫⑮⑯⑰⑲	Modulating oxidative stress
Yuan et al. (2023)	Mouse (C57BL/6J; Male; 6–8 weeks; 18–22 g)	5/5	Tracheotomy injection, BLM 5 mg/kg	NM	100 mg/kg 28 d	①②⑤⑥⑨⑩⑰	Preventing cellular senescence and EMT.
Zheng et al. (2024)	Mouse (C57BL/6J; Male; 6–8 weeks; 18–20 g)	8/8	Intratracheal injection BLM 1.5 U/kg	NM	Inhalation 25 mg/kg, every 3–4 days; 19 days	①②⑦⑧⑨⑰⑱	Inhibition of inflammation and collagen deposition

Abbreviations: AS-IV, Astragaloside IV; BLM, bleomycin; EMT, epithelial-mesenchymal transition; HYP, hydroxyproline; IL-1β, interleukin-1β; IL-6, interleukin-6; IL-18, interleukin-18; M, model group number; MDA, malondialdehyde; NM, not mentioned; ROS, reactive oxygen species; SOD, superoxide dismutase; T, test group number; TGF-β1, transforming growth factor-β1; TNF-α, tumor necrosis factor-α; α-SMA, α-smooth muscle actin.

Note: Numbers in red indicate outcomes that increased after AS-IV, treatment; numbers in blue indicate outcomes that decreased. The numbers represent the following outcome measures: ① α-SMA; ② collagen type I; ③ collagen type I mRNA; ④ collagen type III; ⑤ E-cadherin; ⑥ HYP, content; ⑦ IL-18; ⑧ IL-1β; ⑨ IL-6; ⑩ lung index; ⑪ lung wet/dry weight ratio; ⑫ MDA; ⑬ pulmonary fibrosis score; ⑭ pulmonary inflammation score; ⑮ ROS; ⑯ SOD; ⑰ TGF-β1; ⑱ TNF-α; ⑲ TGF-β1 mRNA.

### 3.2 Assessment of risk of bias

**FIGURE 2:**
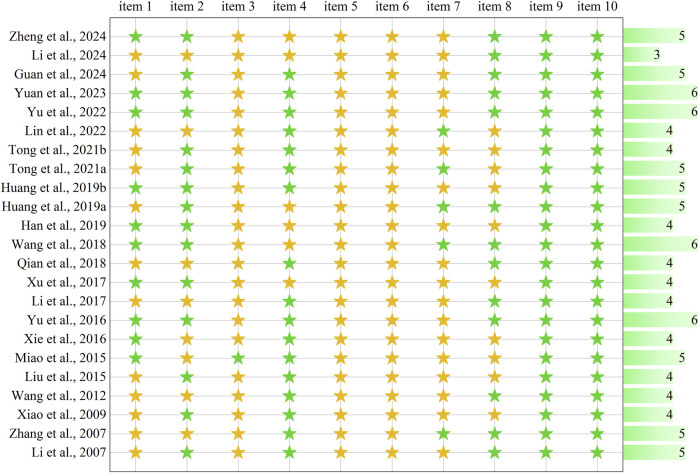
Risk-of-bias and quality assessment scores in each included study. The assessment comprises 10 items: item 1: sequence generation; item 2: baseline characteristics; item 3: allocation concealment; item 4: random housing; item 5: blinding of caregivers/investigators; item 6: random outcome assessment; item 7: blinding of outcome assessor; item 8: incomplete outcome data; item 9: selective outcome reporting; item 10: other sources of bias. Green stars indicate low risk of bias (“yes” response), yellow stars indicate unclear risk due to insufficient reporting (“unclear” response). The horizontal bar graph on the right displays the total number of low-risk items (green stars) for each study, ranging from 3 to 6 out of 10 possible items.

Risk of bias assessment using the SYRCLE tool revealed variable methodological quality across the 23 included studies (Figure 2). Green indicators denoted a low risk of bias, while yellow indicators reflected unclear risk due to insufficient reporting. Notably, 53.5% of all assessed domains were classified as “unclear,” highlighting prevalent reporting deficiencies in the animal literature. Although composite scoring is discouraged given the differential importance of individual bias domains, the mean number of low-risk criteria fulfilled was 4.5 (range: 3–6) out of 10 possible items, which suggests moderate methodological rigor overall, with substantial room for improvement in experimental design and reporting standards within preclinical research on PF.

### 3.3 Effectiveness

#### 3.3.1 Primary outcomes

##### 3.3.1.1 Effect of AS-IV on pulmonary fibrosis score

**FIGURE 3:**
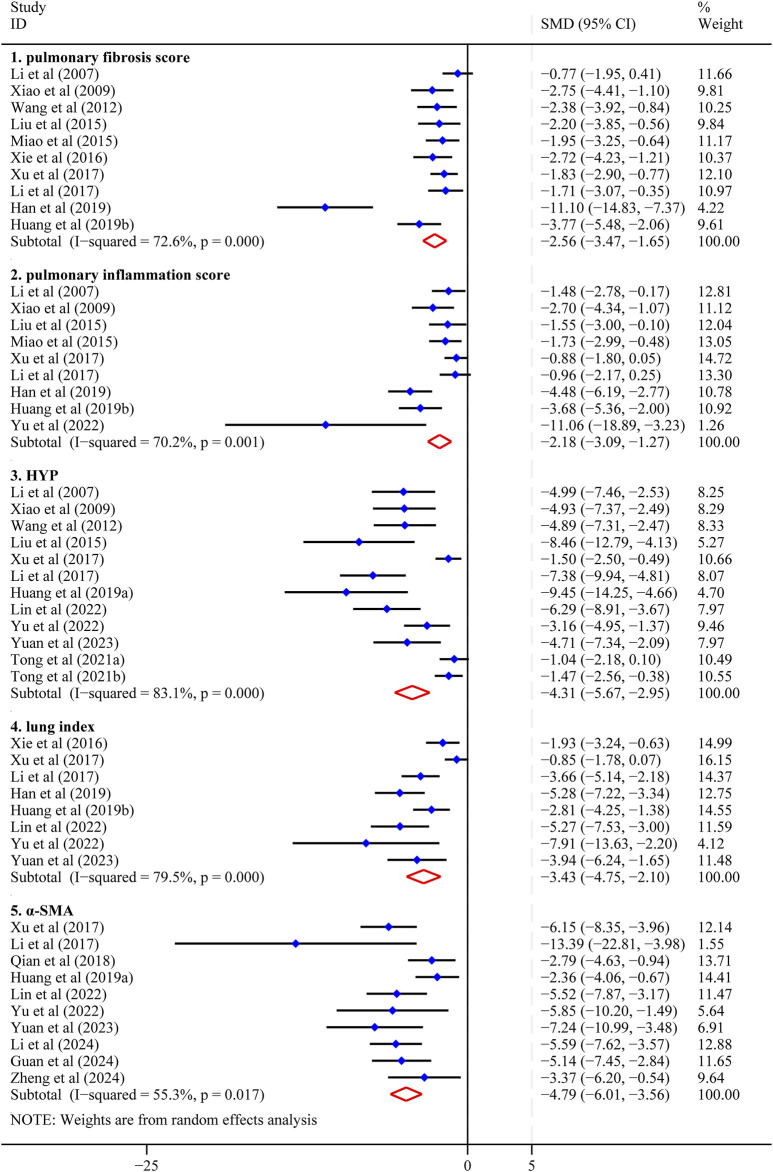
Forest plot (effect size and 95%CI) summarizing the effect of Astragaloside IV on five primary outcome parameters. 1: pulmonary fibrosis score; 2: pulmonary inflammation score; 3: hydroxyproline (HYP); 4: lung index; 5: α-smooth muscle actin (α-SMA).

Ten studies comprising 142 animals were included in the meta-analysis evaluating the effects of AS-IV on PF score. Heterogeneity assessment revealed substantial inter-study variability (*I* ^*2*^ *=* 72.6%; *P <* 0.1 for Q-test), necessitating the application of a random-effects model. The pooled analysis demonstrated that AS-IV treatment significantly reduced PF score compared to control groups [SMD = −2.56, 95% CI (−3.47, −1.65), *P <* 0.01] (Figure 3). This reduction in fibrosis scores suggests that AS-IV may attenuate the pathological remodeling processes characteristic of PF, including excessive collagen deposition and architectural distortion of lung parenchyma.

##### 3.3.1.2 Effect of AS-IV on pulmonary inflammation scores

Nine studies comprising 122 animals were included in the meta-analysis examining the effects of AS-IV on pulmonary inflammation scores. Heterogeneity assessment revealed substantial inter-study variability (*I* ^*2*^ *=* 70.2%; *P <* 0.01 for Q-test), necessitating the application of a random-effects model.

The pooled analysis demonstrated that AS-IV treatment significantly reduced pulmonary inflammation scores compared to control groups [SMD = −2.18, 95% CI (−3.09, −1.27), *P <* 0.01] (Figure 3). This finding suggests that AS-IV has anti-inflammatory effects in lung tissue, potentially mitigating the inflammatory cascade associated with the progression of PF.

##### 3.3.1.3 Effect of AS-IV on HYP content

Fourteen studies evaluating the effects of AS-IV in reducing HYP content in animal models of PF were included in this meta-analysis. The pooled analysis demonstrated that AS-IV treatment significantly reduced HYP levels [SMD = −4.73, 95% CI (−6.52, −2.94), *P <* 0.01] (Supplementary Figure S1), supporting its potential antifibrotic properties.

Further examination identified two studies with markedly divergent characteristics. The studies by Han et al. (2019) and Zhang et al. (2007) exhibited effect sizes that deviated substantially from the other included studies, accompanied by extensive confidence intervals that reflected high statistical uncertainty. These studies contributed minimally to the pooled estimate, with relative weights of 0.15% and 0.00%, respectively. Given their high uncertainty and potential to introduce bias into the pooled results, these studies were excluded from the primary analysis. Sensitivity analysis using the leave-one-out method confirmed the stability of our findings, demonstrating that the exclusion of any single research had minimal impact on the overall effect estimate (Supplementary Figure S2). Following the exclusion of the two outlier studies, the revised pooled effect size remained robust [SMD = −4.31, 95% CI (−5.67, −2.95), *P <* 0.01] (Figure 3), with a reduction in heterogeneity to *I* ^*2*^ *=* 83.1%. The consistency between the original and revised effect estimates, evidenced by overlapping confidence intervals, confirms that these excluded studies had limited influence on the overall meta-analytic conclusions regarding AS-IV’s efficacy in reducing HYP content in PF models.

##### 3.3.1.4 Effect of AS-IV on lung index

Eight studies, comprising 121 animals, were included in the meta-analysis to evaluate the effects of AS-IV on the lung index. Heterogeneity assessment revealed substantial inter-study variability (*I* ^*2*^ *=* 79.5%; Q-test *P <* 0.1), necessitating the application of a random-effects model for data synthesis. The pooled analysis demonstrated that AS-IV treatment significantly reduced lung index compared to control groups [SMD = −3.43, 95% CI (−4.75, −2.10), *P <* 0.01] (Figure 3). This finding suggests a protective effect of AS-IV against pulmonary edema or inflammation, as reflected by the decreased lung index values.

##### 3.3.1.5 Effect of AS-IV on α-SMA

Eleven studies were included to evaluate the effects of AS-IV on α-SMA expression. Heterogeneity assessment revealed substantial inter-study variability (*I* ^*2*^ *=* 82.2%; Q-test *P <* 0.1), suggesting significant heterogeneity across the included studies (Higgins et al., 2003). To identify potential sources of heterogeneity, we conducted a sensitivity analysis using the leave-one-out method. This analysis demonstrated that excluding the study by Han et al. (2019) resulted in a more precise confidence interval and a marked reduction in heterogeneity, with the *I* ^*2*^ statistic decreasing from 82.2% to 55.3% (Supplementary Figure S3). The robustness of our findings was confirmed by the minimal change in the summary effect size following this exclusion. Before exclusion, the pooled effect size was [SMD = −5.08, 95% CI (−7.10, −3.01), *P <* 0.01] (Supplementary Figure S4). Following exclusion, the effect size remained substantially similar at SMD = −4.79 (95% CI: −6.01 to −3.56; *P <* 0.01) (Figure 3). The overlapping confidence intervals between these estimates indicate that the excluded study had minimal impact on the overall meta-analytic findings, supporting the stability of our conclusions regarding AS-IV’s inhibitory effect on α-SMA expression.

#### 3.3.2 Secondary outcomes

##### 3.3.2.1 Impact on EMT biomarkers

Nine studies evaluated the effects of AS-IV on TGF-β1 expression in PF models. Meta-analysis of these studies revealed that AS-IV treatment was associated with reduced TGF-β1 protein levels compared to control groups [n = 124, SMD = −4.43, 95% CI (−6.22, −2.65)]. However, substantial heterogeneity was observed among studies (*I* ^*2*^ *=* 86.5%, *P <* 0.01), suggesting considerable variability in experimental conditions or methodological approaches.

Four studies examined the impact of AS-IV on TGF-β1 mRNA expression. Initial heterogeneity assessment revealed considerable variability between studies (*I* ^*2*^ *=* 89.5%, *P <* 0.01 for the Q-test). To identify potential sources of heterogeneity, we conducted a leave-one-out sensitivity analysis. This analysis indicated that the exclusion of the Lin (2022) study reduced heterogeneity (*I* ^*2*^ decreased from 89.5% to 59.1%). Given concerns regarding methodological quality and its contribution to heterogeneity, this study was excluded from the final analysis. The revised meta-analysis suggested that AS-IV may downregulate TGF-β1 mRNA expression [n = 51, SMD = −1.45, 95% CI (−2.47, −0.43)], though moderate residual heterogeneity persisted (*I* ^*2*^ *=* 59.1%, *P =* 0.087).

**FIGURE 4:**
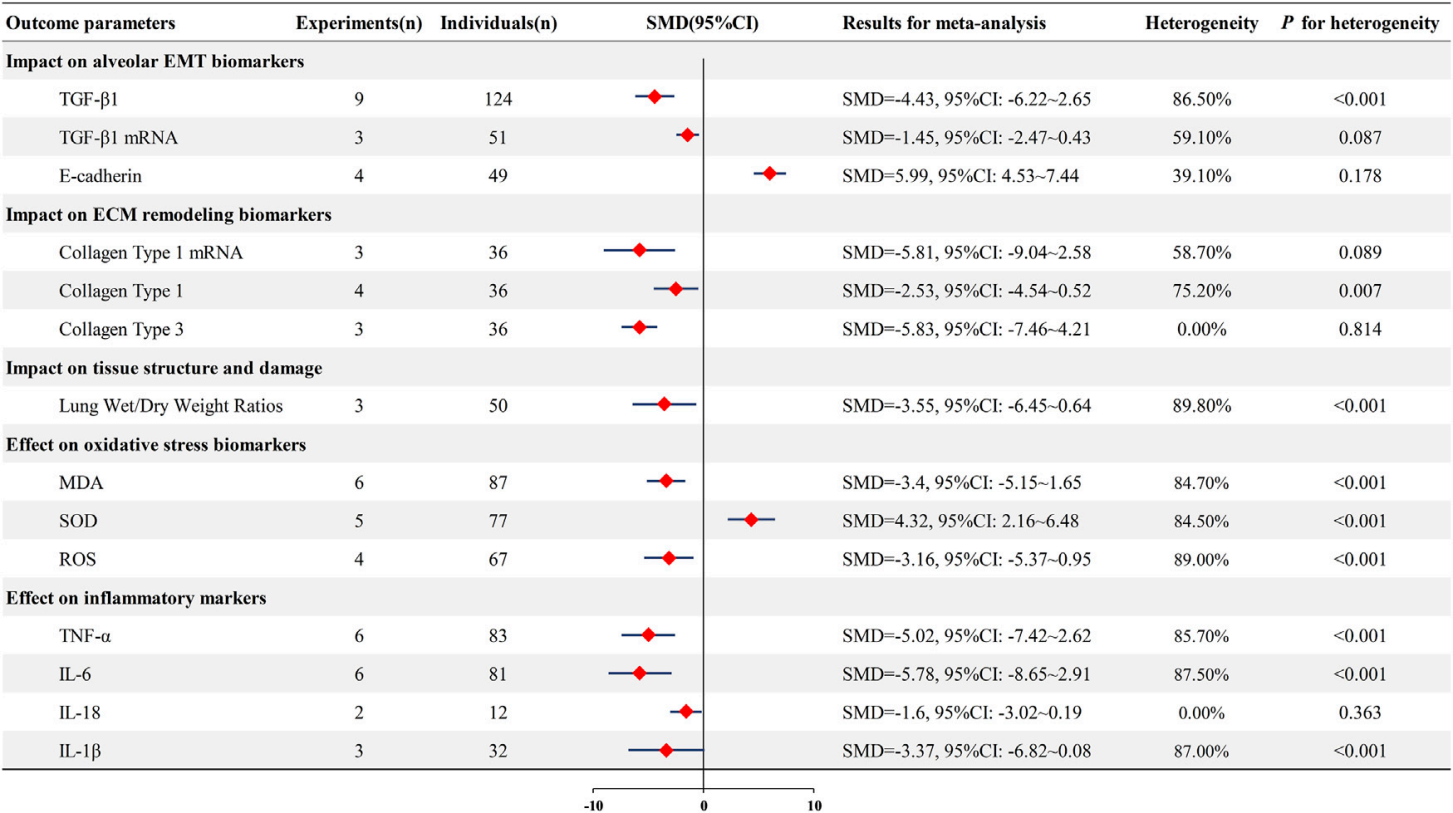
Forest plot (effect size and 95%CI) summarizing the effect of Astragaloside IV on secondary outcome parameters. EMT: epithelial-mesenchymal transition; transforming growth factor-beta 1: TGF-β1; malondialdehyde: MDA; superoxide dismutase: SOD; reactive oxygen species: ROS; tumor necrosis factor-alpha: TNF-α; interleukin-6: IL-6; interleukin-18: IL-18; interleukin-1 beta: IL-1β.

Five studies investigated the effects of AS-IV on E-cadherin expression levels. Initial analysis revealed substantial heterogeneity (*I* ^*2*^ *=* 89.4%, *P <* 0.01). Sensitivity analysis identified the Han et al. (2019) study as a potential contributor to this heterogeneity. Exclusion of this study reduced *I* ^*2*^ from 89.4% to 39.1%, suggesting possible methodological differences or reporting inconsistencies. Following exclusion, meta-analysis of the remaining four studies (n = 49) indicated that AS-IV treatment was associated with increased E-cadherin expression [SMD = 5.99, 95% CI (4.53, 7.44)], with low to moderate heterogeneity (*I* ^*2*^ *=* 39.1%, P = 0.178). These findings are illustrated in Figure 4.

The observed associations between AS-IV treatment and changes in TGF-β1 and E-cadherin expression provide preliminary evidence that AS-IV may influence EMT pathways in PF models. However, the limited number of studies, persistent heterogeneity, and exclusion of studies due to quality concerns necessitate cautious interpretation. These findings suggest the need for additional well-designed studies with standardized experimental protocols to confirm these preliminary observations.

##### 3.3.2.2 Effects on ECM remodeling

Limited data from three studies examined the effects of AS-IV on collagen type 1 mRNA expression in PF animal models. The pooled analysis suggested that AS-IV treatment was associated with reduced collagen type 1 mRNA levels [n = 36, SMD = −5.81, 95% CI (−9.04, −2.58)], though moderate heterogeneity was observed (*I* ^*2*^ *=* 58.7%, *P =* 0.089). Four studies investigated collagen type 1 protein levels following AS-IV treatment. The meta-analysis indicated that AS-IV administration was associated with decreased collagen type 1 protein expression [n = 36, SMD = −2.53, 95% CI (−4.54, −0.52)]. However, substantial heterogeneity was present among these studies (*I* ^*2*^ *=* 75.2%, *P <* 0.01), suggesting variability in experimental protocols or measurement techniques. Three studies evaluated the impact of AS-IV on the expression of collagen type 3. The pooled results indicated that AS-IV treatment was associated with reduced collagen type 3 levels [n = 36, SMD = −5.83, 95% CI (−7.46, −4.21)]. Notably, these studies demonstrated excellent homogeneity (*I* ^*2*^ *=* 0%, *P =* 0.814), indicating consistent findings across the included investigations.

These preliminary findings are illustrated in Figure 4. While the limited number of studies and small sample sizes warrant cautious interpretation, the available evidence suggests that AS-IV may influence collagen deposition in experimental models of PF. The observed heterogeneity, particularly for collagen type 1 assessments, highlights the need for additional studies with standardized methodological approaches to validate these initial observations.

##### 3.3.2.3 Effect of AS-IV on oxidative stress markers

Six studies evaluated MDA levels in PF animal models. Meta-analysis suggested that AS-IV treatment was associated with reduced MDA levels [n = 87, SMD = −3.40, 95% CI (−5.15, −1.65); heterogeneity: *I* ^*2*^ *=* 84.7%, *P <* 0.01]. Five studies assessed SOD activity. The pooled analysis indicated that AS-IV administration was associated with increased SOD levels [n = 77, SMD = 4.32, 95% CI (2.16, 6.48); heterogeneity: *I* ^*2*^ *=* 84.5%, *P <* 0.01]. Five studies examined ROS levels. Meta-analysis suggested that AS-IV treatment was associated with decreased ROS levels [n = 67, SMD = −3.16, 95% CI (−5.37, −0.95); heterogeneity: *I* ^*2*^ *=* 89%, *P <* 0.01].

These findings are depicted in Figure 4. While the limited data suggest that AS-IV may modulate oxidative stress markers in experimental PF, the consistently high heterogeneity across all parameters warrants cautious interpretation and highlights the need for additional standardized studies.

##### 3.3.2.4 Effect of AS-IV on inflammatory cytokines

Meta-analysis indicated that AS-IV was associated with reduced TNF-α levels [n = 83, SMD = −5.02, 95% CI (−7.42, −2.62); heterogeneity: *I* ^*2*^ *=* 85.7%, *P <* 0.01] and decreased IL-6 expression [n = 81, SMD = −5.78, 95% CI (−8.65, −2.91); heterogeneity: *I* ^*2*^ *=* 87.5%, *P <* 0.01]. Limited data from two studies suggested reduced IL-18 levels [n = 12, SMD = −1.60, 95% CI (−3.02, −0.19); heterogeneity: *I* ^*2*^ *=* 0%, *P =* 0.363], while three studies examining IL-1β showed a trend toward reduction that did not reach statistical significance [n = 32, SMD = −3.37, 95% CI (−6.82, 0.08); heterogeneity: *I* ^*2*^ *=* 87%, *P <* 0.01]. These findings are displayed in Figure 4. The substantial heterogeneity observed for most inflammatory markers, combined with the limited number of studies for IL-18 and IL-1β, warrants cautious interpretation of these preliminary results.

##### 3.3.2.5 Effect of AS-IV on lung wet/dry weight ratio

Three studies examined the lung wet/dry weight ratio in PF animal models following AS-IV treatment. A meta-analysis of these limited data indicated that AS-IV administration was associated with a reduced lung wet/dry weight ratio [n = 50, SMD = −3.55, 95% CI (−6.45, −0.64)]. However, substantial heterogeneity was observed among the included studies (*I* ^*2*^ *=* 89.8%, *P <* 0.01) (Figure 4). Given the small number of studies and high heterogeneity, these preliminary findings require cautious interpretation and warrant validation through additional standardized investigations.

### 3.4 Sensitivity analysis

To assess the impact of individual studies on the overall effect size and heterogeneity, we conducted a sensitivity analysis. Specifically, we sequentially excluded each study and observed the changes in effect size and heterogeneity levels. In the analysis of HYP content, the studies by Han et al. (2019) and Zhang et al. (2007) exhibited wide confidence intervals, which could be attributed to high data variability or small sample sizes, leading to unstable results. To ensure the accuracy and stability of the overall effect, we decided to exclude these two studies. The analysis of α-SMA levels and E-Cadherin expression by Han et al. (2019) significantly influenced the *I* ^*2*^ value, resulting in high heterogeneity. Therefore, to improve the accuracy and stability of the analysis, we excluded this study from our analysis. Similarly, the analysis of TGF-β1 mRNA expression by Lin (2022) also significantly impacted the *I* ^*2*^ value, resulting in high heterogeneity. As a result, this study was also excluded. For the remaining outcome measures, sensitivity analysis revealed that excluding individual studies resulted in minimal changes to the effect size and heterogeneity levels, indicating that the results for these outcomes were robust and that the exclusion of any single study had a limited impact on the overall conclusion. After excluding the studies above, a subsequent sensitivity analysis confirmed that both the overall effect and heterogeneity levels remained stable, further supporting the robustness of the conclusions drawn from this study (Supplementary Figure S4).

### 3.5 Subgroup analysis

To address the substantial between-study heterogeneity observed in our analysis, we performed systematic subgroup analyses following established meta-analytical guidelines to identify and characterize potential sources of variation. We comprehensively evaluated heterogeneity across all primary outcome measures—including pulmonary inflammation scores, PF score, HYP content, α-SMA levels, and lung index—stratified by animal species and treatment duration.

The subgroup analyses revealed that animal species contributed significantly to the observed heterogeneity in four outcome measures: PF score, pulmonary inflammation scores, lung index, and HYP content. For α-SMA levels, both animal species and treatment duration emerged as significant sources of heterogeneity, suggesting that these factors independently influence the magnitude of treatment effects across studies (Supplementary Figure S5).

### 3.6 Dose-response relationships and treatment duration analysis

Dose-response relationships and time-efficacy profiles represent critical parameters for optimizing clinical treatment regimens. This study systematically evaluated the impact of AS-IV dosing and treatment duration on antifibrotic efficacy in PF models. By integrating dose and duration data from included studies, we generated a three-dimensional scatter plot (Supplementary Figure S6) that comprehensively illustrates the complex interrelationships among primary outcome measures, treatment duration, and administered doses, while simultaneously considering statistical significance and effect sizes. This advanced visualization approach provides intuitive evidence for understanding the intricate dose-duration-effect relationships, enabling researchers to identify key determinants of therapeutic efficacy. By integrating multidimensional data into a single visual representation, this analytical framework facilitates hypothesis generation and guides the design of future dose-optimization studies, ultimately advancing the translation of AS-IV from preclinical investigation to clinical application.

### 3.7 Publication bias

Publication bias was systematically evaluated for PF score (10 studies), HYP content (14 studies), and α-SMA level (11 studies) using multiple complementary approaches. Visual inspection of funnel plots was initially performed to identify potential asymmetry, followed by statistical testing using Begg’s rank correlation test and Egger’s linear regression test. A significance threshold of *P* ≤ 0.1 was applied for both tests, considering the relatively low statistical power of publication bias detection methods. Statistical testing revealed evidence of potential publication bias across all three outcomes, necessitating the adjustment for bias using the trim-and-fill method.

The trim-and-fill method was employed to estimate the number of potentially missing studies and adjust for funnel plot asymmetry if such asymmetry was due to publication bias. For the PF score, the algorithm imputed three hypothetical studies on the side of the funnel plot with fewer observations, suggesting the presence of publication bias. Despite this adjustment, the corrected pooled effect size remained statistically significant, indicating that the observed therapeutic benefit was not entirely attributable to selective publication. For HYP content and α-SMA level, no missing studies were imputed by the algorithm, and the pooled effect estimates remained unchanged. These findings demonstrate the robustness of the observed treatment effects across all measured outcomes, even when accounting for potential publication bias through conservative statistical adjustment (Supplementary Figure S7).

The meta-analysis demonstrated that AS-IV significantly reduces PF score, HYP content, and α-SMA level. While statistical evidence suggests potential publication bias, sensitivity analysis indicates that this bias does not substantially alter the direction or statistical significance of the observed effects. These findings support the therapeutic potential of AS-IV in conditions associated with fibrosis. However, the presence of publication bias necessitates cautious interpretation and underscores the need for additional high-quality studies to confirm these results.

## 4 Discussion

### 4.1 Outcome profile and originality

PF represents a complex pathological process characterized by progressive pulmonary parenchymal remodelingparenchymal remodeling and irreversible fibrotic changes. Disease progression begins with recurrent injury to alveolar epithelial cells, triggering the release of pro-fibrotic mediators, particularly TGF-β, which promotes local inflammation and activates fibroblasts (Moss et al., 2022). During aberrant wound healing, damaged epithelial cells undergo EMT, which facilitates fibroblast-to-myofibroblast differentiation (Stone et al., 2016). Activated myofibroblasts deposit excess ECM, resulting in tissue stiffening and irreversible architectural distortion (Moss et al., 2022). Simultaneously, oxidative stress, characterized by the accumulation of ROS, exacerbates lipid peroxidation and DNA damage, further amplifying fibrosis through crosstalk with the TGF-β signaling pathway (Estornut et al., 2021). Chronic inflammatory environments, sustained by the continuous release of pro-inflammatory cytokines such as IL-1β and TNF-α, maintain the fibrotic milieu and contribute to immune dysregulation (Shen et al., 2021). Ultimately, excessive ECM deposition and alveolar architectural remodeling lead to honeycombing, traction bronchiectasis, and the irreversible loss of lung function (Deng et al., 2020). The disease’s positive feedback mechanisms further reinforce its irreversibility and pose a severe threat to patient health (Parker et al., 2014). Although current pharmacological treatments such as nintedanib and pirfenidone can modestly slow down disease progression, their limited efficacy and significant side effects highlight the urgent need for safer and more effective therapeutic strategies.

Therefore, AS-IV has emerged as a promising therapeutic candidate, exemplifying the established paradigm of natural product drug development. The successful clinical translation of artemisinin and paclitaxel demonstrated that bioactive compounds isolated from traditional medicinal plants can be developed into effective therapeutics through systematic pharmacological investigations (Tu, 2016; Wani et al., 1971). As a chemically defined, standardized monomeric compound, AS-IV offers distinct advantages, including reproducible manufacturing and precise pharmacokinetic characterization, facilitating rational dose optimization and personalized treatment design (Zhang et al., 2020).

Building on this foundation, we conducted a comprehensive systematic review and meta-analysis following the PRISMA guidelines to systematically evaluate the preclinical evidence for AS-IV in the treatment of PF. Through the searches of multiple databases up to 16 August 2024, 23 eligible preclinical studies were identified. Our meta-analysis revealed that AS-IV demonstrated substantial antifibrotic effects at the histopathological level, effectively reducing PF scores, pulmonary inflammation scores, HYP content, lung index, and lung wet/dry weight ratios, while significantly suppressing aberrant α-SMA expression.

Mechanistic analyses further elucidated that these pathological improvements arose from AS-IV’s coordinated modulation of multiple interconnected molecular pathways. AS-IV effectively reduces the pathological deposition of type I and III collagen by downregulating TGF-β1 protein and mRNA expression while simultaneously preserving the alveolar epithelial cell phenotype by maintaining E-cadherin expression, thereby inhibiting EMT, a critical driver of fibrosis progression. Concurrently, AS-IV demonstrates potent anti-inflammatory activity by significantly reducing the expression of key pro-inflammatory cytokines, including TNF-α, IL-1β, IL-6, and IL-18, while alleviating oxidative stress through the upregulation of SOD activity and a reduction in MDA levels and ROS generation. This multidimensional biological activity profile, encompassing antifibrotic, anti-inflammatory, antioxidant, and immunomodulatory effects, enabled AS-IV to simultaneously target multiple critical nodes in the pathogenesis of PF.

The mechanistic insights gained from this meta-analysis not only deepen our understanding of AS-IV’s antifibrotic pharmacology but also provide a robust scientific foundation for clinical translation. The identified pharmacological targets and biomarkers could directly inform endpoint selection and patient stratification strategies in future clinical trials. Following the successful translational pathway established by artemisinin and paclitaxel, this accumulated preclinical evidence suggests that AS-IV, as a multitarget antifibrotic candidate, holds promise for addressing the substantial unmet medical needs in this disease, which is characterized by poor prognoses and severely limited therapeutic options. These findings validate the scientific rationale and feasibility of developing standardized natural products for modern therapeutics.

### 4.2 Potential mechanisms of action

#### 4.2.1 Attenuation of oxidative stress

Oxidative stress, characterized by excessive ROS production, is a critical pathological mechanism in PF progression (Estornut et al., 2021; Sul et al., 2022). ROS contribute to disease pathogenesis through multiple mechanisms: direct damage to alveolar epithelial cells, activation of the TGF-β signaling pathway, and perpetuation of the pro-fibrotic microenvironment (Otoupalova et al., 2020). Excessive ROS levels induce mitochondrial dysfunction and DNA damage, disrupt cellular homeostasis, and accelerate fibrotic processes (Li et al., 2020). Consequently, the modulation of oxidative stress represents a promising therapeutic approach for protecting lung tissue from progressive injury and reducing the development of fibrosis.

Our meta-analysis revealed that AS-IV treatment was associated with reduced ROS and MDA levels and increased superoxide dismutase activity in experimental PF models. These findings suggest that AS-IV may alleviate redox imbalances by modulating both the pro-oxidant and antioxidant systems. The observed reduction in oxidative stress markers may contribute to a decrease in ROS-mediated cellular toxicity and potentially attenuate TGF-β pathway activation. Supporting evidence from diabetic nephropathy models suggests that AS-IV can enhance mitochondrial function and mitigate oxidative damage by upregulating the Nrf2-ARE/TFAM signaling pathway (Shen et al., 2023), potentially indicating mechanistic parallels in PF.

Based on these preliminary findings, AS-IV may exert antifibrotic effects through complementary mechanisms: enhancing mitochondrial function to reduce ROS generation and modulating TGF-β/Smad signaling to disrupt pro-fibrotic processes. However, the substantial heterogeneity observed across studies, combined with the limited number of investigations, necessitates cautious interpretation. Future research should focus on elucidating the precise molecular mechanisms underlying AS-IV’s antioxidant effects in PF, standardizing experimental protocols, and evaluating its translational potential in well-designed clinical studies.

By targeting the interplay between oxidative stress and the TGF-β signaling pathway, AS-IV may exert its effects through a dual mechanism: improving mitochondrial function to mitigate ROS toxicity and suppressing the excessive activation of the TGF-β/Smad signaling pathway, thereby disrupting the stability of the fibrotic microenvironment. These findings highlight the significant potential of AS-IV in modulating oxidative stress and protecting lung tissue, while also suggesting its capacity to alleviate fibrosis progression through synergistic multi-pathway effects. Further investigation of its precise mechanisms and clinical applications is essential to comprehensively evaluate its therapeutic value in PF.

#### 4.2.2 Suppression of inflammatory responses

Chronic inflammation promotes the irreversibility of PF by sustaining the overexpression of profibrotic cytokines and disrupting immune homeostasis (Janho Dit Hreich et al., 2024). Studies have shown that key inflammatory mediators such as TNF-α, IL-1β, IL-6, and IL-18 play pivotal roles in maintaining the inflammatory microenvironment and exacerbating fibrosis (Borthwick, 2016). Lung tissue injury induces the release of these mediators that activate immune responses and establish a positive feedback loop between inflammation and fibrosis, thereby accelerating fibrosis progression (Wynn and Ramalingam, 2012).

These inflammatory mediators drive the progression of inflammation and fibrosis through multiple signaling pathways. For instance, IL-1β and TNF-α activate the nuclear factor-kappa B (NF-κB) signaling pathway, thereby enhancing the expression of pro-inflammatory cytokines and amplifying the inflammatory response (Yu et al., 2020). IL-6 modulates immune cell activation through the JAK/STAT3 signaling pathway, thereby sustaining a chronic inflammatory state and exacerbating fibrosis (Li Y. et al., 2022). Furthermore, IL-18 activates both NF-κB and MAPK signaling pathways through a MyD88-dependent mechanism, promoting immune cell infiltration and the release of pro-inflammatory cytokines, thereby accelerating the progression of fibrosis (Janho Dit Hreich et al., 2024; Landy et al., 2024).

Our study demonstrated that AS-IV significantly reduced the levels of pro-inflammatory mediators, including TNF-α, IL-1β, IL-6, and IL-18, in experimental models while markedly improving pulmonary inflammation scores. By inhibiting these key inflammatory factors, AS-IV disrupts the positive feedback loop between inflammation and fibrosis, restores immune homeostasis, and mitigates the progression of inflammation-driven fibrosis. This mechanism suggests that AS-IV exerts antifibrotic effects by targeting inflammation, highlighting its potential as a multitarget therapeutic agent. These findings underscore the clinical translational value of AS-IV as a promising candidate for the treatment of inflammation-driven fibrosis and warrant further investigation.

#### 4.2.3 Inhibition of EMT

EMT is a complex biological process that plays a critical role in various pathological conditions, particularly in the onset and progression of PF (Rout-Pitt et al., 2018). EMT is characterized by the loss of epithelial cell polarity and cell-cell adhesion, accompanied by the acquisition of mesenchymal traits, including enhanced migratory and invasive capacities, as well as increased production of ECM (Jayachandran et al., 2021). Studies have shown that in fibrotic lung regions, damaged alveolar epithelial cells undergo EMT and transform into fibroblasts and myofibroblasts. These cells secrete large amounts of collagen and other ECM proteins, leading to tissue stiffening and gradual loss of normal lung function (Willis and Borok, 2007). Moreover, EMT disrupts the epithelial barrier integrity, facilitating the diffusion of inflammatory and fibrosis-associated factors within the tissue, thereby stabilizing the fibrotic microenvironment. Thus, EMT is recognized as a key irreversible mechanism that drives PF.

TGF-β1 is a central regulatory factor in EMT and the progression of PF, with elevated mRNA and protein expression levels representing key features of early-stage fibrosis (Evrard et al., 2012). By binding to TGF-β receptors, TGF-β1 activates the Smad signaling pathway, inducing the phosphorylation of Smad2/3 (Budi et al., 2021). This signaling cascade suppresses the expression of the epithelial marker E-cadherin while upregulating mesenchymal markers such as fibronectin and vimentin, thereby initiating the EMT process (Wu et al., 2018). E-cadherin is a critical marker of EMT, and its high-level expression in epithelial cells is essential for maintaining tissue integrity and cell polarity (Seo et al., 2019). Downregulation of E-cadherin disrupts cell-cell adhesion, promotes the transition of epithelial cells into mesenchymal-like fibroblasts and contributes to excessive ECM deposition and stabilization of the fibrotic microenvironment (Ghosh et al., 2022; Peng et al., 2020).

Furthermore, TGF-β1 can enhance the expression of Wnt ligands, such as Wnt3a, via Smad signaling, thereby activating the Wnt/β-catenin signaling pathway (Li et al., 2021). The Wnt/β-catenin pathway upregulates EMT transcription factors, such as Snail and Twist (Zhang et al., 2024), which further suppress E-cadherin expression while enhancing fibroblast migration and ECM accumulation. The synergistic activation of the TGF-β/Smad and Wnt/β-catenin pathways amplifies the pathological processes of EMT and fibrosis, forming a positive feedback loop that promotes ECM synthesis and stabilizes the fibrotic microenvironment (Somanader et al., 2024; Zhao et al., 2022). Thus, elevated TGF-β1 expression and reduced E-cadherin levels represent two critical nodes in EMT and fibrosis progression that are closely interlinked and mutually reinforced.

Our study demonstrates that AS-IV significantly inhibits EMT and mitigates the progression of fibrosis through multiple mechanisms. Specifically, AS-IV markedly reduces the mRNA and protein expression levels of TGF-β1, thereby attenuating the activity of the TGF-β/Smad signaling pathway. Concurrently, AS-IV upregulates E-cadherin expression, which helps restore epithelial cell adhesion properties and blocks EMT initiation. These findings suggest that AS-IV disrupts the core pathological processes of EMT and fibrosis progression by modulating TGF-β1 and E-cadherin-associated signaling molecules, effectively alleviating the pathological progression of PF. This study highlights the potential of AS-IV as a multitarget anti-fibrotic candidate, warranting further preclinical and clinical research.

#### 4.2.4 Regulation of ECM remodeling

The ECM plays a critical role in maintaining tissue structural integrity and regulating cellular functions. Its primary components include collagen, elastin, glycosaminoglycans, fibronectin, and laminins (Theocharis et al., 2019). In PF, ECM remodeling is a central pathological process characterized by excessive ECM deposition and impaired ECM degradation. These alterations disrupt the lung tissue architecture, reduce elasticity, and impair gas exchange, thereby driving disease progression. Studies have shown that abnormal ECM remodeling is closely associated with the overactivation of the TGF-β/Smad signaling pathway, with activated myofibroblasts serving as the primary effector cells responsible for excessive ECM deposition (Meng et al., 2015).

α-SMA is a classical marker of myofibroblast activation, and its upregulation is typically associated with the excessive secretion of type I and III collagens (Herum et al., 2013). As the major components of fibrotic scar tissue, type I and type III collagens play crucial roles in maintaining ECM stiffness and structural integrity (Mu et al., 2010). However, excessive accumulation significantly increases lung tissue rigidity and contributes to impaired PF (Qian et al., 2018). Additionally, HYP, a key marker of collagen metabolism, directly reflects increased ECM synthesis and severity of fibrosis when elevated (Ricard-Blum et al., 2018). Therefore, dynamic changes in α-SMA, type I and III collagen, and HYP levels are critical indicators for assessing the extent of fibrosis.

Our study demonstrated that AS-IV significantly reduced the expression level of α-SMA, the transcription level of type I collagen mRNA, and the protein expression levels of type I and III collagens, as well as the HYP levels. These findings suggest that AS-IV mitigates ECM overdeposition by inhibiting myofibroblast activation, thereby partially restoring the ECM metabolic balance. Based on these results, we hypothesized that AS-IV may attenuate the pathological progression of PF by targeting the key processes involved in ECM remodeling. This discovery provides preliminary evidence for further investigation of its precise mechanisms and preclinical applications.

In summary, AS-IV exhibited robust antifibrotic properties by targeting oxidative stress, inflammation, EMT, and ECM remodeling, rendering it a promising therapeutic candidate for PF. We further performed subgroup analyses of the PF score, pulmonary inflammation scores, lung coefficients, HYP levels, and α-SMA levels. The results indicated that animal species might be the primary source of heterogeneity in PF score, pulmonary inflammation scores, lung coefficients, and HYP levels. Additionally, both animal species and treatment duration were identified as potential contributors to the heterogeneity in α-SMA levels. However, owing to the limited sample size, these findings should be interpreted with caution, and further high-quality studies are needed to provide more robust evidence.

### 4.3 Limitations and considerations

While this meta-analysis provides valuable insights into the therapeutic potential of AS-IV in PF, several limitations should be acknowledged along with the strengths of our systematic approach and comprehensive analysis.

#### 4.3.1 Study limitations and heterogeneity considerations

The primary limitations of this study stem from the substantial heterogeneity among the included studies. First, we observed marked variability in the AS-IV administration protocols. The included studies exhibited significant heterogeneity in treatment initiation times (ranging from days 2, 3, 8, or 15 post-BLM induction), dosage ranges (1–200 mg/kg), administration frequencies (from once every 3–4 days to daily), routes of administration (predominantly oral gavage, supplemented by inhalation and intratracheal administration), and treatment durations (14–28 days). This methodological diversity potentially contributes to the variations in therapeutic outcomes and biomarker responses. Second, the heterogeneity in animal models is a significant challenge. The included studies utilized two rodent species (47.8% rats and 52.2% mice) across multiple strains (mice: C57BL/6J, Kunming, and C57BL/6; rats: Sprague-Dawley and Wistar). Notably, genetic background substantially influences both fibrotic susceptibility and pharmacological responses (Walkin et al., 2013), introducing biological variability that may confound meta-analytic conclusions. Furthermore, substantial variations in the pathological parameters and biomarker measurements across studies may have influenced the reliability and generalizability of our findings. To address these heterogeneity concerns, we implemented three analytical strategies: (1) application of random-effects models to accommodate inter-study variance, (2) subgroup analyses based on treatment durations (28 days vs <28 days), and (3) implementation of species-level subgroup analyses. However, a limited number of studies have precluded more granular subgroup analyses examining the timing of treatment initiation, administration routes, or specific animal strains. Although random-effects models effectively manage the inherent heterogeneity in preclinical studies (Hooijmans et al., 2014), they cannot eliminate the influence of biological variations. Notably, sensitivity analyses, including the exclusion of high-risk-of-bias studies and trim-and-fill analyses to address publication bias, consistently demonstrated the robust therapeutic effects of AS-IV across diverse experimental conditions. These findings not only strengthen the evidence for AS-IV’s therapeutic potential but also underscore the critical need for comprehensive methodological improvements in preclinical PF research. Specifically, we advocate three key initiatives: (1) standardization of experimental protocols to ensure reproducibility and comparability across studies (Begley and Ioannidis, 2015); (2) implementation of core outcome sets to facilitate meaningful meta-analyses and evidence synthesis (Clarke and Williamson, 2016; Williamson et al., 2017); and (3) enhancement of reporting standards and data transparency to minimize publication bias and maximize research utility (Moher et al., 2016). Such systematic improvements are essential to accelerate the translation of promising therapeutic candidates from preclinical studies to clinical applications.

#### 4.3.2 Publication bias and language considerations

In our analysis, we identified potential publication bias through funnel plot asymmetry and Egger’s test, which is a well-recognized challenge in preclinical meta-analyses in which negative results are less frequently published (Sena et al., 2010). We acknowledge that preferential publication of positive results may lead to inflated effect estimates (Sterne et al., 2011). Considering these limitations, we emphasize that our findings should be interpreted with appropriate caution. Notably, the trim-and-fill analysis demonstrated that our primary conclusions remained robust despite these constraints. To address this issue in future research, we advocate for prospective registration of animal studies, which could substantially mitigate publication bias and enhance the reliability of preclinical evidence synthesis (Bert et al., 2019).

Among the 23 included studies, 14 (60.9%) were published in Chinese journals, reflecting substantial research interest in TCM-derived compounds within their cultural origins. While this distribution may raise concerns about language bias and limited international accessibility, our risk of bias assessment revealed no significant differences in methodological quality between the English- and Chinese-language publications. Both groups demonstrated comparable distributions of low-, unclear-, and high-risk ratings across all bias domains. This finding corroborates previous systematic assessments showing that publication language is not necessarily correlated with study quality in research (Moher et al., 1996). The predominance of Chinese literature is understandable, given that AS-IV is derived from AM, a traditional Chinese medicinal herb with an extensive research infrastructure in China (Li et al., 2014). However, this geographical concentration of the research may influence the generalizability of the findings and introduce regional methodological variations. Despite our unrestricted search strategy, the inclusion of studies published in only two languages remains a limitation. Future systematic reviews should aim to overcome this by incorporating broader international collaboration and multilingual search capabilities. This limitation highlights the need for more diverse and internationally coordinated research to validate these findings across different research settings and populations (Dirnagl et al., 2013).

#### 4.3.3 Sample size limitations and statistical power considerations

Our primary efficacy endpoints demonstrated adequate statistical power, with the number of studies ranging from eight comparisons for lung index to 12 comparisons for HYP content. However, the statistical power of the secondary endpoints exhibited considerable heterogeneity across the biomarker categories. Moderately powered analyses were achieved for the pro-fibrotic cytokine TGF-β1 at the protein level (n = 9 studies), inflammatory mediators TNF-α and IL-6 (n = 6 each), and oxidative stress biomarkers including SOD activity, MDA levels, and ROS (n = 5–6). In contrast, several mechanistically critical endpoints demonstrated limited statistical power: the pro-inflammatory cytokines IL-18 (n = 2) and IL-1β (n = 3), transcriptional markers (TGF-β1 mRNA and Collagen Type I mRNA, n = 3 each), extracellular matrix components (Collagen Type I protein, n = 4; Collagen Type III protein, n = 3), and the pulmonary edema indicator (lung wet-to-dry weight ratio, n = 3). These constrained sample sizes, particularly for endpoints with n ≤ 3, introduce substantial limitations: wide confidence intervals, reduced power to detect heterogeneity through *I* ^*2*^ statistics (Higgins et al., 2003), inability to conduct meaningful sensitivity analyses or meta-regression (Fu et al., 2008), and precluded assessment of publication bias through funnel plot asymmetry (Sterne et al., 2011). Despite these statistical power constraints, we retained these secondary outcomes in our analysis to provide a comprehensive evaluation of AS-IV’s multifaceted antifibrotic mechanisms. Although individual biomarker results based on limited studies should be interpreted with appropriate caution, the consistent directionality of the effects across all analyzed biomarkers, including those with smaller sample sizes, provides preliminary evidence of AS-IV’s anti-inflammatory and antioxidant properties. This inclusive approach, although admittedly limited by the sample size for specific endpoints, enabled a more comprehensive assessment of AS-IV’s therapeutic profile and generated hypotheses for future targeted investigations. We emphasize that the conclusions regarding these secondary biomarkers should be considered preliminary and require validation through additional well-designed studies with adequate sample sizes. We emphasize that the conclusions regarding these secondary biomarkers should be considered preliminary and require validation through additional well-designed studies with sufficient statistical power.

#### 4.3.4 Translational challenges and future directions

All included studies evaluated short-term outcomes (≤28 days), precluding the assessment of sustained efficacy or delayed adverse effects. The long-term safety profile and durability of the antifibrotic effects remain undetermined, limiting the clinical applicability of AS-IV for PF, a chronic, progressive disease requiring extended treatment (Lederer and Martinez, 2018). Furthermore, the predominant use of early intervention protocols in animal models, with treatment typically initiated within 1 day post-induction, contrasts sharply with clinical reality, where diagnosis is often delayed and intervention occurs at advanced stages of the disease (Lamas et al., 2011). This temporal discrepancy, combined with the absence of subgroup analyses based on different PF models (e.g., idiopathic vs secondary PF), limits our understanding of specific pathological mechanisms and may hinder the identification of patient subgroups most likely to benefit from AS-IV therapy. The well-documented limitations of translating findings from BLM-induced rodent models to heterogeneous human PF subtypes further complicate clinical translation (Jenkins et al., 2017). Future studies should incorporate delayed intervention models to simulate clinical scenarios better and provide more translatable evidence (Tashiro et al., 2017).

These constraints highlight the inherent challenges of extrapolating preclinical findings to clinical practice. Future investigations should prioritize standardized protocols, clinically relevant intervention timings, and extended treatment durations to generate more translatable evidence. Addressing these limitations through a rigorous study design is essential for determining AS-IV’s therapeutic potential in human PF and for developing TCM-derived antifibrotic agents.

## 5 Conclusion

This systematic review and meta-analysis provide robust evidence that AS-IV ameliorates BLM-induced PF through multiple mechanisms, including the suppression of inflammation, attenuation of oxidative stress, inhibition of ECM deposition, and modulation of EMT. The therapeutic effects demonstrated in 23 preclinical studies (n = 518 animals) were consistent despite methodological heterogeneity, supporting AS-IV’s potential as a multitarget antifibrotic agent.

Nevertheless, translating these encouraging preclinical findings into clinical practice requires careful consideration of several factors. The inherent limitations of the current studies, including methodological heterogeneity, variations in treatment protocols, and the predominance of early intervention models, underscore the need for more standardized and clinically relevant research approaches. Future investigations should prioritize the development of optimized dosing regimens through rigorous pharmacokinetic and pharmacodynamic studies, implementation of delayed intervention protocols that better reflect clinical scenarios, and direct comparisons with established antifibrotic therapies. Additionally, extending treatment duration beyond the current 28-day maximum is crucial for evaluating long-term efficacy and safety profiles. Biomarker identification may facilitate patient stratification and the development of personalized treatment approaches.

The urgent need for novel antifibrotic therapies, combined with AS-IV’s favorable safety profile in preclinical studies, underscores the importance of its continued development as a potential therapeutic candidate. The mechanistic insights and effective dose ranges identified in this meta-analysis provide a rational foundation for future translational studies. However, the path from preclinical efficacy to clinical benefit remains unclear, necessitating carefully designed trials with appropriate biomarkers and functional endpoints to evaluate AS-IV’s therapeutic potential in human PF.

## Data Availability

The original contributions presented in the study are included in the article/Supplementary Material, further inquiries can be directed to the corresponding author.

## Glossary

PF: pulmonary fibrosis
AS-IV: Astragaloside IV
AM: Astragalus mongholicus
TCM: Traditional Chinese Medicine
BLM: bleomycin
HYP: Hydroxyproline
TGF-β1: Transforming growth factor-β1
α-SMA: α-smooth muscle actin
EMT: Epithelial-mesenchymal transition
ECM: Extracellular matrix
CNKI: China National Knowledge Infrastructure
WanFang: Wan Fang Data Knowledge Service Platforms
CQVIP: China Science and Technology Journal Database
CBM: China Biological Medicine Database
P: Population
I: Intervention
C: Control
O: Outcome
N: Number
95%CI: 95% Confidence Interval
SMD: Standardized Mean Difference
SD: Standard Deviations
I2: I-squared
NM: Not mentioned
MDA: Malondialdehyde
ROS: Reactive oxygen species
SOD: Superoxide dismutase
IL-6: Interleukin-6
IL-18: Interleukin-18
IL-1β: Interleukin-1β
TNF-α: Tumor necrosis factor-α
NF-κB: Nuclear factor-kappa B
PRISMA: Preferred Reporting Items for Systematic Reviews and Meta-analyses
SYRCLE: SYstematic Review Centre for Laboratory animal Experimentation.
