# Supplementary figures and images for: Therapeutic effects and potential mechanisms of astragaloside IV on pulmonary fibrosis: a systematic review and meta-analysis of preclinical studies
Source: Front Pharmacol. 2025 Jul 31;16:1564290. doi: 10.3389/fphar.2025.1564290 (PMC12350305; doi:10.3389/fphar.2025.1564290)

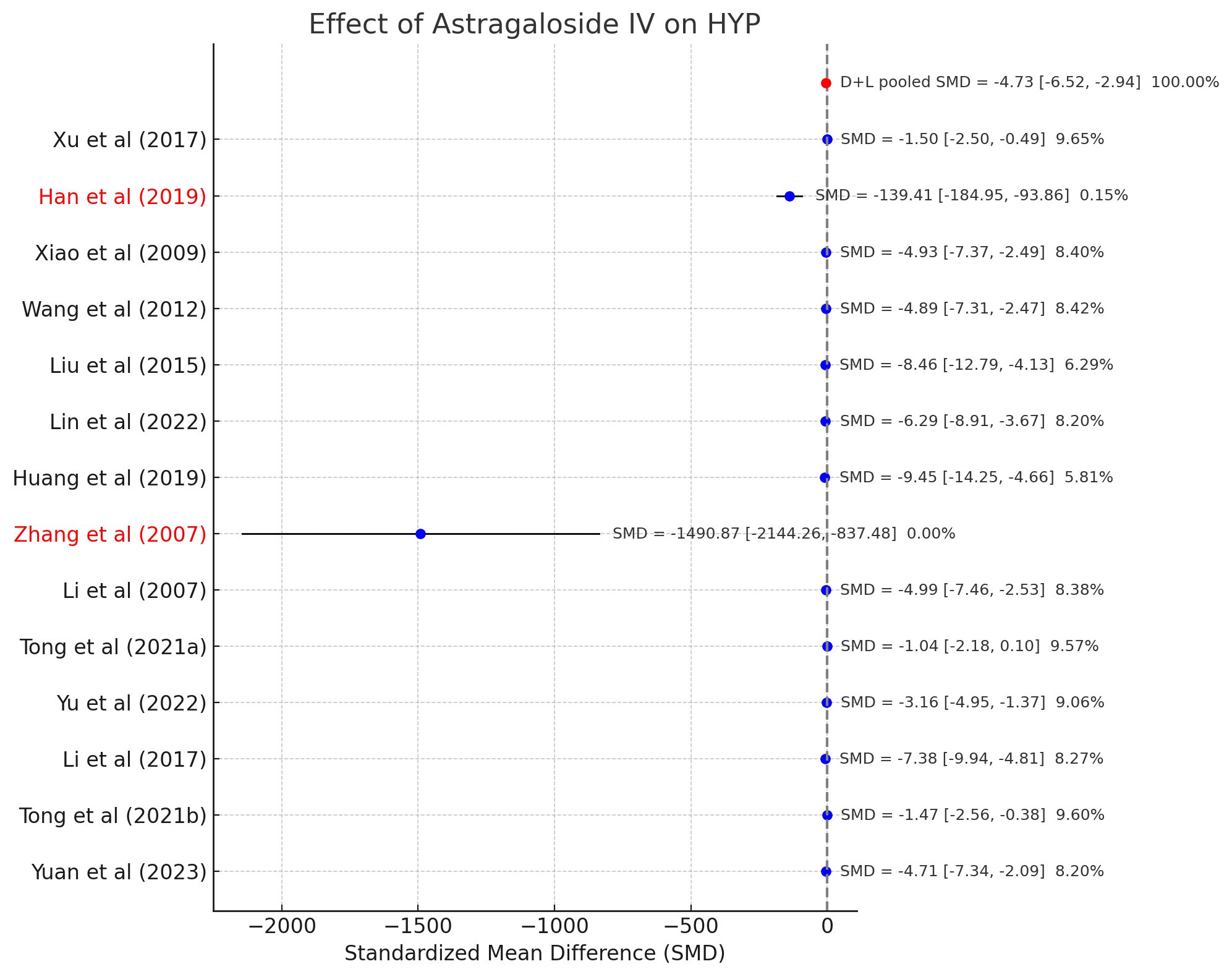

Supplement: Supplementary file 1 [file Image1.tiff]

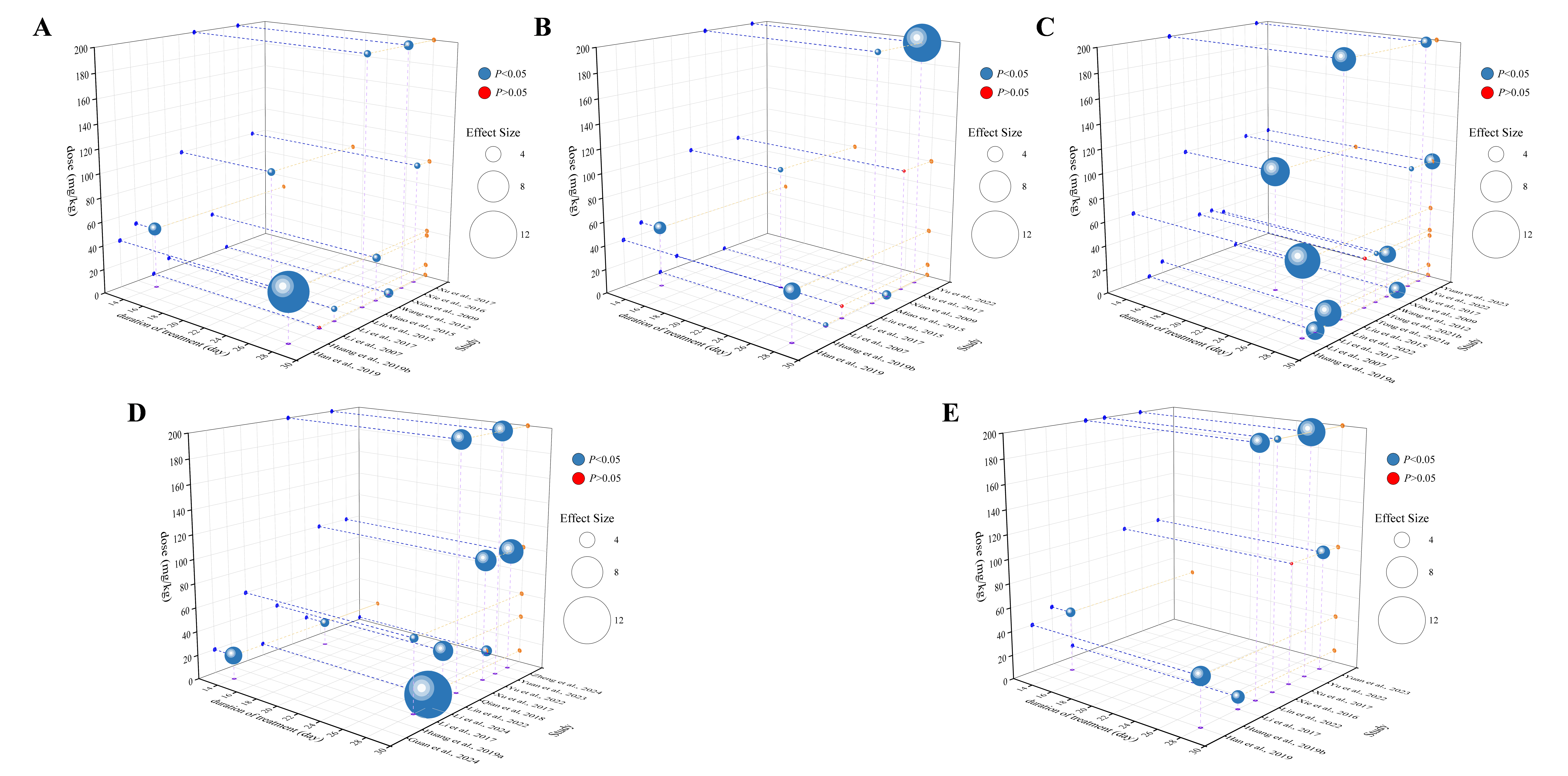

Supplement: Supplementary file 2 [file Image6.tif]

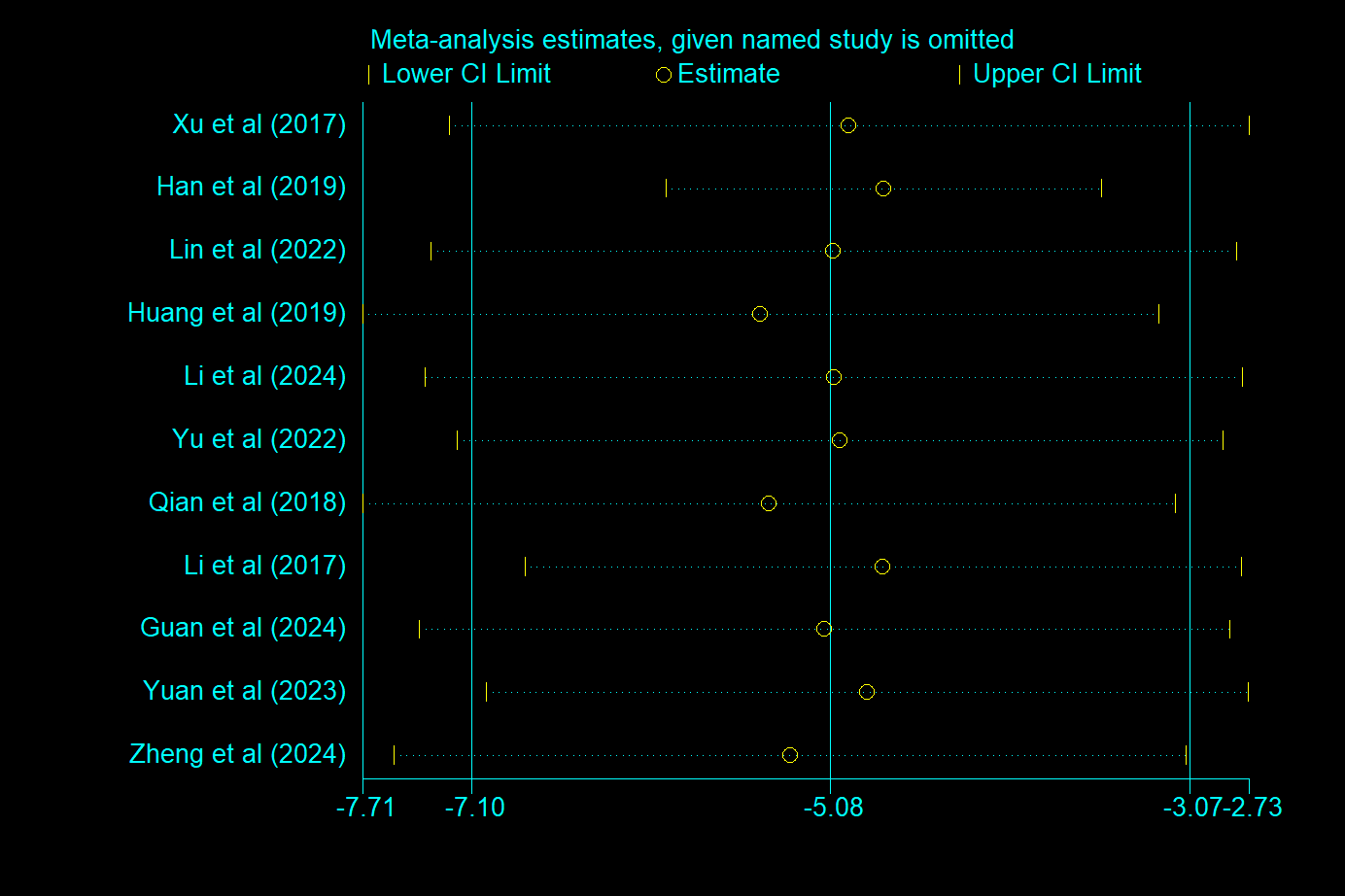

Supplement: Supplementary file 3 [file Image3.tif]

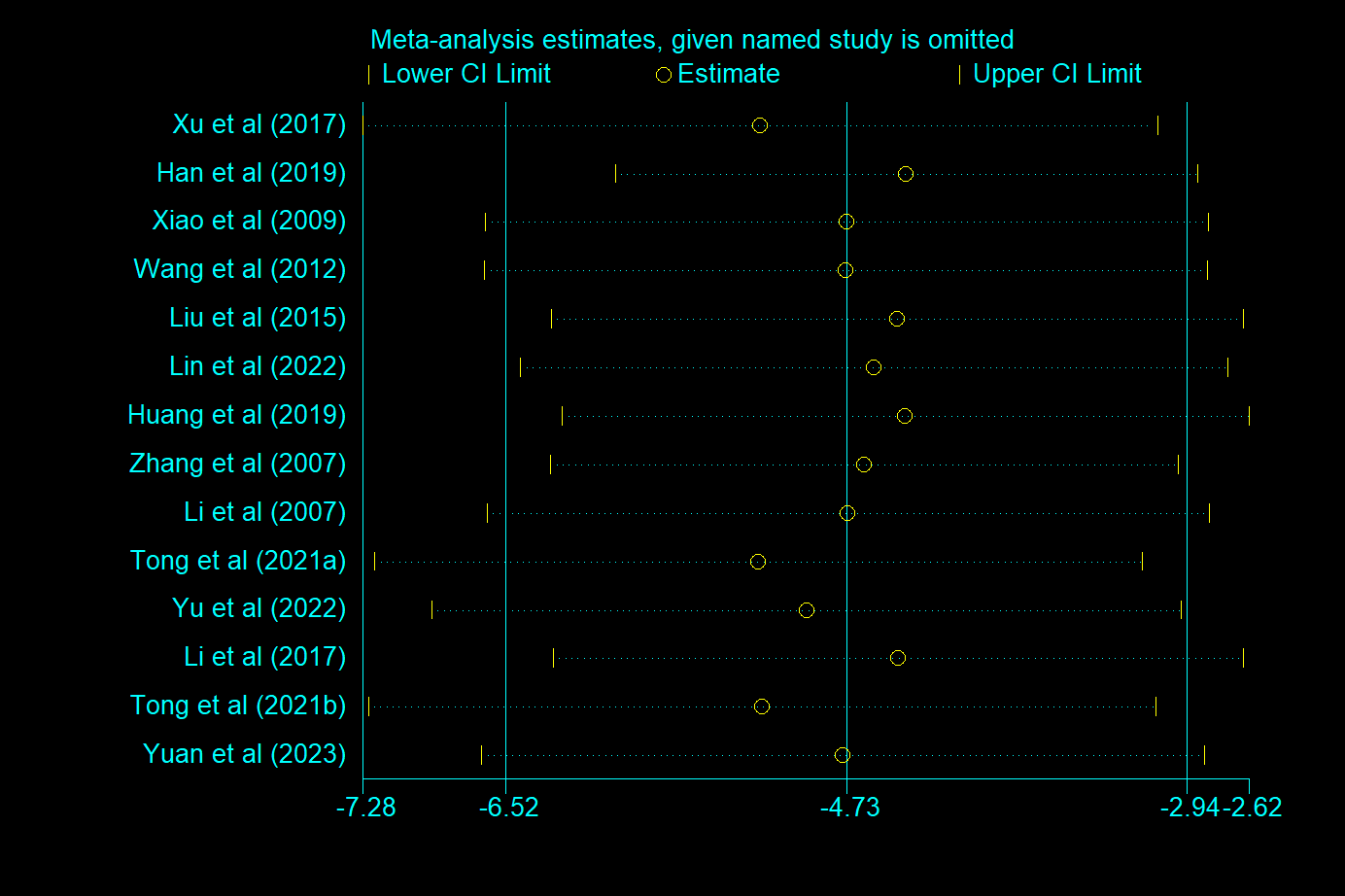

Supplement: Supplementary file 6 [file Image2.tif]
