# Supplementary material for: Therapeutic effects and potential mechanisms of astragaloside IV on pulmonary fibrosis: a systematic review and meta-analysis of preclinical studies
Source: Front Pharmacol. 2025 Jul 31;16:1564290. doi: 10.3389/fphar.2025.1564290 (PMC12350305; doi:10.3389/fphar.2025.1564290)
Supplement: Supplementary file 8 [file Table1.doc]

## Supplementary Table 1: Search Strategy in PubMed

| **Search** | **Query** | **Items found** |
| --- | --- | --- |
| #1 | Pulmonary Fibrosis[MeSH] | 29732 |
| #2 | (((((((Fibroses, Pulmonary[Title/Abstract]) OR (Fibrosis, Pulmonary[Title/Abstract])) OR (Pulmonary Fibroses[Title/Abstract])) OR (Alveolitis, Fibrosing[Title/Abstract])) OR (Alveolitides, Fibrosing[Title/Abstract])) OR (Fibrosing Alveolitides[Title/Abstract])) OR (Fibrosing Alveolitis[Title/Abstract])) OR (Idiopathic Diffuse Interstitial Pulmonary Fibrosis[Title/Abstract]) | 51962 |
| #3 | #1OR#2 | 60591 |
| #4 | "astragaloside A" [Supplementary Concept] | 807 |
| #5 | ((((astragaloside-A[Title/Abstract]) OR (astragaloside IV[Title/Abstract])) OR (astramembrannin I[Title/Abstract])) OR (cyclosiversioside F[Title/Abstract])) OR (3beta,6alpha,16beta,20R,24S; astragaloside IV of astragaloside A[Title/Abstract]) | 1148 |
| #6 | #4OR#5 | 1232 |
| #7 | #3 AND #6 | 22 |

## **Supplementary Table 2: List of Abbreviations**

| **Abbreviation** | **Full Name** |
| --- | --- |
| 95%CI | 95% Confidence Interval |
| AM | Astragalus mongholicus |
| AS-IV | Astragaloside IV |
| BLM | bleomycin |
| C | Control |
| CBM | China Biological Medicine Database |
| CNKI | China National Knowledge Internet |
| CQVIP | China Science and Technology Journal Database |
| ECM | extracellular matrix |
| EMT | epithelial-mesenchymal transition |
| HYP | Hydroxyproline |
| I | Intervention |
| I² | I-squared |
| IL-18 | Interleukin-18 |
| IL-1β | Interleukin-1 beta |
| IL-6 | Interleukin-6 |
| MDA | Malondialdehyde |
| N | Number |
| NF-κB | Nuclear factor-kappa B |
| NM | Not mentioned |
| P | Population |
| PF | pulmonary fibrosis |
| PRISMA | Preferred Reporting Items for Systematic Reviews and Meta-analyses |
| ROS | Reactive oxygen species |
| SD | Standard Deviation |
| SMD | Standardized Mean Difference |
| SOD | Superoxide dismutase |
| TCM | Traditional Chinese Medicine |
| TGF-β1 | Transforming growth factor-β1 |
| TNF-α | Tumor necrosis factor-α |
| WanFang | WanFang Data Knowledge Service Platform |
| α-SMA | α-Smooth muscle actin |
